# Naphthalene: irritative and inflammatory effects on the airways

**DOI:** 10.1007/s00420-020-01636-0

**Published:** 2021-01-19

**Authors:** Kirsten Sucker, Wolfgang Zschiesche, Mohammed Aziz, Tanja Drews, Thomas Hummel, Monika Raulf, Tobias Weiss, Daniel Bury, Dietmar Breuer, Silke Werner, Claudia Friedrich, Jürgen Bünger, Dirk Pallapies, Thomas Brüning

**Affiliations:** 1 Institute for Prevention and Occupational Medicine of the German Social Accident Insurance (IPA), Bochum, Germany; 2 Department of Otorhinolaryngology, Interdisciplinary Center “Smell and Taste”, TU Dresden, Dresden, Germany; 3 Institute for Occupational Safety and Health of the German Social Accident Insurance (IFA), Sankt Augustin, Germany

**Keywords:** Naphthalene, Acute and chronic effects, Sensory irritation, Inflammation, Human study

## Abstract

**Objective:**

This cross-sectional study determined whether acute sensory irritative or (sub)chronic inflammatory effects of the eyes, nose or respiratory tract are observed in employees who are exposed to naphthalene at the workplace.

**Methods:**

Thirtynine healthy and non-smoking male employees with either moderate (*n* = 22) or high (*n* = 17) exposure to naphthalene were compared to 22 male employees from the same plants with no or only rare exposure to naphthalene. (Sub)clinical endpoint measures included nasal endoscopy, smell sensitivity, self-reported work-related complaints and the intensity of naphthalene odor and irritation. In addition, cellular and soluble mediators in blood, nasal lavage fluid (NALF) and induced sputum (IS) were analysed. All measurements were carried out pre-shift on Monday and post-shift on Thursday. Personal air monitoring revealed naphthalene shift concentrations up to 11.6 mg/m^3^ with short-term peak concentrations up to 145.8 mg/m^3^ and 1- and 2-naphthol levels (sum) in post-shift urine up to 10.1 mg/L.

**Results:**

Acute sensory irritating effects at the eyes and upper airways were reported to occur when directly handling naphthalene (e.g., sieving pure naphthalene). Generally, naphthalene odor was described as intense and unpleasant. Habituation effects or olfactory fatigue were not observed. Endoscopic examination revealed mild inflammatory effects at the nasal mucosa of exposed employees in terms of reddening and swelling and abnormal mucus production. No consistent pattern of cellular and soluble mediators in blood, NALF or IS was observed which would indicate a chronic or acute inflammatory effect of naphthalene in exposed workers.

**Conclusions:**

The results suggest that exposure to naphthalene induces acute sensory irritative effects in exposed workers. No (sub)chronic inflammatory effects on the nasal epithelium or the respiratory tract could be observed under the study conditions described here.

## Introduction

Naphthalene (CAS-Nr. 91-20-3) is a white crystalline solid that evaporates at room temperature, has a characteristic tar-like odour, and can be smelled at concentrations as low as 0.44 mg/m^3^ (0.084 ppm) (Amoore and Hautala 1983). Naphthalene is mainly used for the synthesis of phthalic anhydride. However, the use of naphthalene as a pore-forming agent in the manufacture of abrasives is an important niche application. There, naphthalene is used openly rather than in closed production cycles. Naphthalene is also a component of tobacco smoke and a residue in tar-containing building products.

Exposure to naphthalene at the workplace or via the environment occurs primarily via inhalation. As sson as naphthalene is taken up, it is mainly metabolized to 1- and 2-naphthol by cytochrome P450 (CYP) monooxygenases and various dihydroxynaphthalenes and quinones.

A wide variety of effects of naphthalene have been previously described. For example, local P450 metabolism in the airways and formation of electrophilic metabolites followed by activation of transient receptor potential ankyrin 1 channels (TRPA1) on trigeminal nerve endings is a potential pathway how naphthalene can elicit sensory irritation responses in the upper airways and the eyes (Chiu et al. 2012; Lanosa et al. 2010). Naphthalene might also induce neurogenic inflammation which is supposed to be a physiological sign of the transition from a reversible stimulation of the trigeminal nerve fibers to an adverse health effect (Brüning et al. 2014). Because trigeminal nerve endings are able to release neuropeptides (e.g., Substance P) that regulate immune cell responses and are also equipped with cytokine receptors (e.g., IL-1β, TNF-α receptors) that can respond to inflammatory mediators, prolonged exposures to naphthalene might also trigger responses from the immune system. This kind of bidirectional neuro-immune crosstalk has been recently described for pain and inflammatory diseases (Pinho-Ribeiro et al. 2017).

Currently, there is inadequate evidence in humans for the carcinogenicity of naphthalene and it is grouped in category 2B by the International Agency for Research on Cancer of the WHO (IARC 2002). However, naphthalene causes tumors in the nose and lung of rodents after inhalation (NTP 1992, 2000). The respiratory and olfactory epithelia of the nose have been found to be particularly sensitive. Essentially, cytotoxic effects and chronic inflammatory reactions have been suggested as decisive factors for the observed carcinogenic effects in the respiratory tract of rats and mice (Bailey et al. 2016). Although the relevance of naphthalene-induced tumors in rodents remain largely unclear in humans due to considerable anatomical and physiological differences (Morris and Shusterman 2010), the basic mechanism by which naphthalene causes these tumors in rodents (chronic inflammation of the respiratory tract) is also valid in humans.

To study whether acute and (sub)chronic local irritant and possible inflammatory effects on the nasal epithelium in humans can be observed after occupational exposure to naphthalene we investigated workers in five production plants of the abrasive industry. This collective has been chosen because naphthalene is used openly in the abrasive industry and, at the same time, the workplaces are characterized by low co-exposures to other workplace contaminants thus limiting the influence of potential confounding factors on health outcome. These assumptions have been confirmed specifically for our collective. In example, previously published data on exposure to naphthalene in the collective presented here (Weiss et al. 2020) revealed high naphthalene shift concentrations up to 11.6 mg/m^3^ with short-term peak concentrations up to 145.8 mg/m^3^ (e.g., sieving pure naphthalene). In addition, biological monitoring revealed 1- and 2-naphthol concentrations in post shift urine up to 10.1 mg/L. In contrast, past measurements by air sampling in three out of the five companies suggested only low exposures to dust with the inhalable fraction ≤ 5.5 mg/m^3^ and the respirable fraction ≤ 1.0 mg/m^3^ (unpublished company data). In addition, in two out of the five companies concentrations of crystalline silica up to 100 µg/m^3^ have been measured.

Here, we report the work-related complaints about odor annoyance and eye, respiratory and mucous membrane irritation during work, and the odor perception and sensory irritation before and after work. (Sub)clinical signs of irritation, inflammation and damage to the nasal mucosa were investigated by nasal endoscopy, whereas smell sensitivity was assessed by the Sniffin’ Sticks test. Furthermore, changes in humoral and cellular compositions of blood, nasal lavage fluid (NALF) and induced sputum (IS) were examined.

## Material and methods

### Study design and subjects

**Fig. 1:**
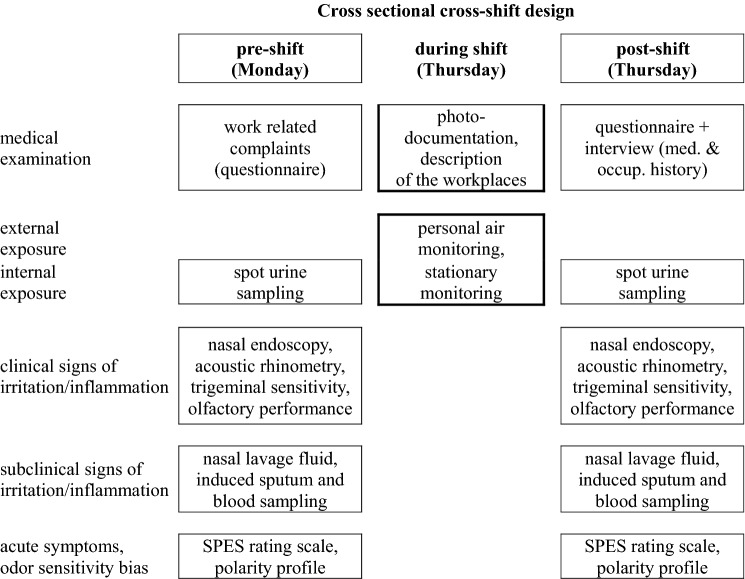
Cross-sectional cross-shift design of the Naphthalene study

A cross-sectional and a cross-week design has been chosen (Fig. 1) by assessing health complaints and effects with a preference on sensory irritation and nasal inflammation in 61 employees of 5 companies (Germany: 3; Austria: 2) of the abrasive industry on Monday pre-shift and Thursday post-shift. Overall, the study design offered the possibility to assess acute effects post-shift on Thursday and (sub)chronic effects pre-shift on Monday.

Based on previously published data on exposure assessment in air, results on biological monitoring, and workplace description and working history, the participants could be divided into a highly (*n* = 22) and a moderately exposed (*n* = 17) group to naphthalene, and a reference group (*n* = 22) (Weiss et al. 2020). The highly exposed group included employees directly exposed to naphthalene while mixing, sieving, moulding, or pressing naphthalene containing formulations. The moderately exposed participants worked in post-processing areas which were located in close proximity to workstations with open handling of naphthalene. The persons in the reference group had not been directly working with naphthalene for the past 10 years and either worked in spatially separated offices or, evidenced by exposure assessment, in post-processing areas with no or miniscule exposures to naphthalene. All investigated persons were male and non-smokers to exclude confounding effects of tobacco smoke, a well-known respiratory irritant. For this purpose, smoking status was pre-assessed by both, questionnaire and cotinine in urine. Persons with urinary cotinine concentrations above 100 µg/L were considered smokers (Haufroid and Lison 1998) and excluded from the study (prior group assignment). Atopy status was determined serologically, testing specific immunoglobulin IgE antibodies in the serum to a variety of environmental allergens (sx1 Phadiatop, Phadia Upsala Sweden).

### Work-related complaints

Self-reported work-related complaints were examined by structured questionnaire pre-shift on Monday. The questionnaire comprised 21 symptoms including both specific irritative effects at the eyes, nose or throat, and non-specific symptoms (e.g., headache). The list of symptoms was chosen from the subtest of the Swedish Performance Evaluation System (SPES, Iregren et al. 1997). Instead of a 6-point rating scale with values ranging from ‘not at all’ (0) to ‘very, very much’ (5) subjects were simply asked “Do you experience the following symptoms at work or immediately afterwards?” (Yes/No). The ratings were combined with those on self-reported complaints on ocular irritation (burning eyes, dry eyes, watering eyes), nasal irritation (itching nose, dry nose, running nose, stuffy nose, frequent sneezing, nosebleeds), pharyngeal symptoms (coughing spells, shortness of breath), and general complaints (headache, dizziness, nausea, and perspiration). To verify whether complaints are work-related or could be attributed to other causes (e.g., asthma, allergies, wearing contact lenses), the study subjects were also asked whether there would be an improvement or absence of complaints after work, the weekend, a vacation, and whether the complaints had also occurred on other occasions.

### Perception ratings of odor and irritation

Chemosensory perception ratings were assessed pre-shift on Monday and post-shift on Thursday by using ‘labeled magnitude scales’ (LMS) (Green et al. 1996). Participants were asked “Please indicate whether and to what extent you currently experience the indicated sensation”. Olfactory descriptors were used to rate different percepts: odor intensity, annoyance, and nausea. Trigeminal descriptors were used to rate eye (burning, tickling) and nasal irritation (‘sneeze’, prickling, sharp, pungent). The LMS rating was administered via personal computer monitor. Each descriptor could be rated by six categories with quasi-logarithmic spacing between each category including ‘barely detectable’ (10), ‘weak’ (55), ‘moderate’ (165), ‘strong’ (355), ‘very strong’ (530), and ‘strongest imaginable’ (1000).

Furthermore, a naphthalene odor sample was evaluated using the polarity profile method (Sucker and Hangartner 2012), also known as semantic differential scaling. The scale consisted of 29 pairs of adjectives (*X*–*Y*) to describe different sensory experiences (e.g., strong–weak, cold–hot, pleasant–unpleasant etc.). Each pair was rated on a 7-point scale with values from − 3 (‘extremely *X*’) via 0 ( *neither X* *nor Y*) to + 3 (‘extremely *Y*’). The obtained profile of the naphthalene odor was compared to established representative profiles of “fragrance” and “stench” (Sucker and Hangartner 2012) to rate the perceived naphthalene odor profile for each subject. To exclude a potential odor sensitivity bias the Chemical Sensitivity Scale (CSS) (Nordin et al. 2003, 2004) was used on Monday pre-shift.

### Otorhinolaryngological examination

The examination of the nose and throat (ENT) was performed Monday pre-shift and Thursday post-shift. Endoscopy of the nasopharyngeal area was performed using a 30° rigid endoscope (Storz^®^, Tuttlingen, Germany) to examine the mucosa on both sides. Recorded images were evaluated by two experts independently and double-blinded, i.e., to the other expert and to the participants’ exposure group. Reddening and swelling of the nasal mucosa and the degree to which the nasal mucus production was serous or purulent was graded from zero to 2 (not present: 0; moderate: 1; pronounced: 2). Results for each nasal side (left vs. right) were first recorded separately and then combined to obtain a “total endoscopy score” that ranged from 0 to 16. The level of inflammation increases with an increasing score (Soler et al. 2016; Poletti et al. 2018).

Smell sensitivity was assessed with the Sniffin’ Sticks odour threshold test (Burghart Medizintechnik, Wedel). For this purpose, n-butanol was presented in 16 increasing concentration steps. Normosmia were defined as a score ≥ 6.75, hyposmia as a score between 1.25 and 6.5, and anosmia by not beeing able to identify the highest concentration (score = 1) (Hummel et al. 1997).

### Inflammatory markers

Blood samples (serum), nasal lavage fluid (NALF) and induced sputum (IS) were collected Monday pre-shift and Thursday post-shift. NALF was obtained as described earlier (Raulf-Heimsoth et al. 2000). IS was collected by inhalation of isotonic (0.9%) saline aerosol, generated by an ultrasonic nebulizer for 10 min. All samples once taken were immediately cooled and sent to IPA, and processed on the same day. The samples were analysed for their composition of cellular and soluble markers to investigate early signs of irritation/inflammation on the systemic (serum) and on the local level such as the nasal mucosa (NALF, IS). The concentration of Club cell secretory protein (CC-16) was determined using a sandwich ELISA from BioVendor (Brno, Czech Republic). In addition to total cell numbers and differential cell counts of NALF and IS cells, concentrations of IL-8, MMP-9, IL-6, TIMP-1, 8-iso-PGF2α and LTB4 were determined by immunoassays based on monoclonal or polyclonal antibodies and according to the recommendations of the manufacturers (Raulf-Heimsoth et al. 2011). C-reactive protein (CRP) was determined in serum samples obtained on Monday pre-shift only.

### Statistical analysis

Statistical evaluation was performed with SPSS (v. 22.0) and Graph Pad Prism (v. 5.04). A non-normal distribution of the data was assumed. Descriptive statistics (arithmetic mean, median, standard deviation, minimum and maximum) were calculated. Depending on the outcome parameter (ordinal scaled, interval-scaled) the Fisher’s exact test and the Kruskal–Wallis test was used to compare the three exposure groups (reference, moderately, highly exposed). To detect changes during the week, both the Mann–Whitney test and ANOVA was applied to compare the pre-shift results on Monday vs. post-shift on Thursday. To calculate the relationship between naphthalene exposure and effect parameters Spearman's rank correlation coefficient (*r*_S_) was used. To assess the relationships between exposure and endpoints of acute irritative and inflammatory, personal shift measurements (air) and 1- and 2-naphthol in post-shift urine on Thursday was used, whereas naphthol levels in pre-shift urine on Monday was used to assess the relationship between exposure and (sub)chronic effects. For evaluation of the polarity profiles, the similarity of the naphthalene odor profile to representative profiles of the concepts of “fragrance” and “stench” was calculated with the Pearson product-moment correlation (*r*). Overall, the significance level (*p* = 0.05) was corrected using the Bonferroni method.

## Results

### Characteristics of the study group

The characteristics of the study groups are summarized in Table 1. The duration of exposure to napththalene was comparable in the two exposure groups (*p* = 0.392). No increased frequency of atopy (*p* = 0.569) or respiratory allergy (*p* = 0.777) in the two exposure groups compared to the references could be observed. The same was true for chronic diseases (*p* = 0.363), nasal diseases (*p* = 0.419), and diseases of the respiratory tract (*p* = 1.000). The three groups differed only slightly in terms of age (*p* = 0.042), i.e., those in the highly exposed group were on average 7 years younger than in the other two groups. The employees in the highly exposed group also described themselves as less sensitive to odors and chemicals than in the other two groups (*p* = 0.006).

**Table 1:** Characteristics of the study groups

	Reference	Moderately exposed	Highly exposed
Beforehand: reference (*n* = 31)	23	8	–
Exposed (*n* = 32)	–	9	23
History-/exposure measurement based (*N* = 63)	23	17	23
Exclusion of two subjects suspected of current smoking (*n* = 2)	22	17	22
Age (years) [mean; median (min–max)]	46; 49 (23–62)	46; 48 (24–60)	39; 41 (25–58)
Duration of exposure (years in this work) [mean; median (min–max)]	9.0; 9.1(0.6–34.4)	9.1; 10.4 (0.4–33.9)	6.8; 7.0 (0.3–21.8)
1 year (number)	2	1	1
Ex-smoker (*n*; %)	11; 50	9; 53	7; 32
Never smoked (*n*; %)	11; 50	8; 47	15; 68
Positive atopy status^a^ (*n*; %)	7; 35^e^	6; 38^f^	11; 50
Allergy (*n*; %)	3; 14	4; 24	5; 23
Chronic disease^b^ (*n*; %)	12; 55	10; 59	8; 36
Disease of the nose^c^ (*n*; %)	6; 27	2; 12	7; 32
Disease of the airways^d^ (*n*; %)	2; 9	2; 12	2; 9
Chemical sensitivity scale (CSS) (median; min–max)	57; 46–74	57; 17–84	50; 32–73

^a^Sensitization to ubiquitous inhalation allergens (determination of specific IgE antibodies using the screen test (sx1); _s_IgE > 0.35 kU/L are seen as positive)

^b^Malfunction of the thyroid gland, migraine, high blood pressure, high cholesterol, others

^c^History of nasal surgery, polyps with medical diagnosis, others

^d^Chronic rhinitis, bronchitis, asthma, others

^e^Only 20 subjects could be tested

^f^Only 16 subjects could be tested

Exposure assessment (air and biological monitoring) in all groups has been previosly outlined in detail (Weiss et al. 2020). In brief, median naphthalene concentrations in air (Thursday) in the highly and moderately exposed group was 6.30 mg/m^3^ and 0.59 mg/m^3^, whereas it was 0.13 mg/m^3^ in the controls and thus well below the former occupational exposure limit of 0.5 mg/m^3^ in Germany (AGS 2011) (Table 2). In individual cases, especially during sieving of pure naphthalene, short-term measurements revealed concentrations up to 145.8 mg/m^3^ in the group of highly exposed workers. Similar differences have been observed for the sum of 1- and 2-naphthol in post-shift urine samples (again Thursday) of the highly (1256 μg/g creatinine) and the moderately exposed group (108 μg/g). In contrast, median exposure was 10 μg/g in controls and thus well below the reference level of the general population in Germany (DFG 2019).

**Table 2:** Air and biological monitoring results: personal shift measurements (mg/m^3^) and sum of 1- and 2-naphthol (μg/g creatinine)

Mean ± SD Median (Min–Max)	Shift measurements (mg/m^3^)	Sum of 1- and 2-naphthol, post-shift (μg/g creatinine)
Reference	0.15 ± 0.10	18 ± 11
	0.13 (0.05–0.36)	10 (6–40)
Moderately exposed	0.66 ± 0.27	108 ± 49
	0.59 (0.20–1.22)	108 (43–210)
Highly exposed	6.97 ± 3.10	1489 ± 999
	6.30 (2.46–11.58)	1256 (293–4352)

### Work-related complaints and perception ratings

Significantly more work-related eye (*p* = 0.001) and nasal complaints (*p* = 0.016) were reported in the highly exposed group (eye: *n* = 14; nose: *n* = 12) compared to the reference group (eye: *n* = 5; nose: *n* = 4). Additionally, a higher percentage of nasal but not eye complaints was also reported in the moderately exposed group (eye: *n* = 2; nose: *n* = 10) compared to the reference group. No differences were found between the groups in terms of pharyngeal complaints (*p* = 0.869) or other more general complaints (e.g., headaches) (*p* = 0.365). Employees in the highly exposed group attributed their eye complaints to the naphthalene exposure, whereas employees in the reference group attributed it to visual display unit (VDU) work. Employees in the highly exposed group further explained that eye and nose irritation were noticeable only when handling naphthalene directly.

Positive correlations between work-related eye and nasal complaints with exposure were observed. In example, the correlation between eye complaints with naphthalene in the air was *r*_S_ = 0.400 (*p* = 0.001; *n* = 61) and with post-shift biomonitoring results *r*_S_ = 0.410 (*p* = 0.001; *n* = 61). A similar result was obtained when the product of exposure duration (years in this work) and naphthalene concentration in air (in mg/m^3^) was used (*r*_S_ = 0.337; *p* = 0.008; *n* = 61) in terms of estimating the cumulative exposure to naphthalene. This evaluation is based on the assumption that the exposure for each study subject is relatively constant over time. For nasal complaints the correlation with the naphthalene concentration in the air was *r*_S_ = 0.290 (*p* = 0.023; *n* = 61), with the post-shift biomonitoring results *r*_S_ = 0.333 (*p* = 0.009; *n* = 61), and with the exposure index *r*_S_ = 0.173 (*p* = 0.182; *n* = 61).

After the end of the shift, generally less complaints were reported albeit no complete absence of complaints could be found (Table 3). Sensory irritation at the nose and eyes, and odor intensity/annoyance were assessed with values between ‘barely detectable’ and ‘weak’. However, seven employees still reported moderate to strong eye or nose irritation. Three employees were even very annoyed by the naphthalene odor and reported ‘strong’ and ‘strongest imaginable’ odor intensity/annoyance.

**Table 3:** Cross-week changes of chemosensory perception ratings, comparison of study groups (median; min–max)

	Time	Reference (*n* = 22)	Moderately exposed (*n* = 17)	Highly exposed (*n* = 22)
Odor intensity	Monday	48 (0–365)	0 (0–158)	0 (0–309)
	Thursday	60 (0–399)	0 (0–153)	114 (0–1000)
Odor annoyance	Monday	14 (0–375)	0 (0–162)	0 (0–264)
	Thursday	21 (0–158)	0 (0–77)	52 (0–1000)
Eye irritation	Monday	15 (0–267)	0 (0–150)	22 (0–191)
	Thursday	15 (0–172)	29 (0–161)	26 (0–256)
Nose irritation	Monday	19 (0–232)	6 (0–99)	19 (0–161)
	Thursday	13 (0–146)	17 (0–78)	39 (0–240)

‘Barely detectable’ (10) through ‘weak’ (55), ‘moderate’ (165), ‘strong’ (355), ‘very strong’ (530), to ‘strongest imaginable’ (1000)

The naphthalene odor was judged as a stench (*r* = 0.93) and the opposite of a fragrance (*r* = − 0.78). The odor was also described as intensive (median 6, range 2–7) and unpleasant (median 6, range 3–7). No differences between the exposure groups were found and no habituation effect to the naphthalene odor during the study week could be identified (values not shown).

### Otorhinolaryngological examination

The endoscopic examination of the nose revealed no (sub)clinical signs of irritation, inflammation or damage of the nasal mucosa on Monday (*p* = 0.365) (Table 4). On Thursday, mild inflammatory effects were observed in the two exposure groups compared to the reference group (reference vs. moderately exposed: *p* = 0.0001; reference vs. highly exposed: *p* = 0.022), whereas no differences were found between the moderately and the highly exposed group (moderately vs. highly exposed: *p* = 0.145). However, the results revealed no dose–response relationship in terms of an increasing number of inflammatory effects or increasing severity with increasing exposure. The effect of measurement time was statistically significant [*F*_(1,57)_ = 22.653, *p* = 0.0001], also the exposure group effect [*F*_(1,57)_ = 4.281, *p* = 0.018], and the interaction effect (*F*_(2,57)_ = 4.490, *p* = 0.015). The correlation between the total endoscopic score measured on Thursday with the corresponding naphthalene concentration in the air was *r*_S_ = 0.340 (*p* = 0.008; *n* = 60), with the post-shift biomonitoring results *r*_S_ = 0.286 (*p* = 0.027; *n* = 60), and with the exposure index *r*_S_ = 0.287 (*p* = 0.026; *n* = 60).

**Table 4:** Cross-week changes of clinical findings of the nasal mucosa (reddening, swelling) und mucus production (purulent, serous) (total endoscopic score), comparison of study groups (median; min–max)

	Time	Reference (*n* = 22)	Moderately exposed (*n* = 17)	Highly exposed (*n* = 22)
Total endoscopic score	Monday	1.5 (0–11)	2.5 (0–12)	3.0 (0–8)
	Thursday	2.5 (0–9)	5.0 (3–13)	4.5 (0–13)

A detailed inspection of the endoscopic scores for reddening or swelling of the nasal mucosa and serous or purulent mucous production showed that, generally, purulent secretion was most often observed. Purulent secretion occurred in half of the moderately (*n* = 9) and highly (*n* = 11) exposed employees, whereas it occurred only in one third of the reference group (*n* = 5). However, purulent secretion was generally at a low level. Altogether, the maximum endoscopic scores were not higher than “2”, with one exception of “3” in the highly exposed group on a scale between 0 (no effects at all) and 16 (in case all four endpoints were present at the highest score of 2 and in both sites of the nose).

When looking at the results of the Sniffin’ Sticks test, only one employee from the reference group exhibited significantly impaired olfactory response (anosmia). A total of 13 employees had a decreased sense of smell (hyposmia). No elevated prevalence of chronically impaired olfactory response was observed in the moderately (normosmia: *n* = 15) or highly exposed (normosmia: *n* = 17) group compared with the reference group (normosmia: *n* = 16) (*p* = 0.695).

### Inflammatory markers in serum, NALF and sputum

No significant differences between exposed groups and the controls could be observed for CC16 in serum neither on Monday before starting work (*p* = 0.452) nor on Thursday after the end of the shift (*p* = 0.074). However, all CC16 values were lower on Thursday post-shift compared to Monday pre-shift (Table 5). This difference was somewhat more pronounced in the highly exposed group (*p* = 0.0001), compared to the reference (*p* = 0.014) and the moderately exposed group (*p* = 0.008). The effect of measurement time was statistically significant [*F*_(1,54)_ = 46.005, *p* = 0.0001], but not the exposure group effect [*F*_(1,54)_ = 1.829, *p* = 0.170], or the interaction effect [*F*_(2,54)_ = 0.382, *p* = 0.685]. The correlation between the CC16 values measured on Thursday and the post-shift biomonitoring results was *r*_S_ = − 0.319 (*n* = 57; *p* = 0.016). The correlation between the CC16 values measured on Thursday and the creatinine-adjusted pre-shift biomonitoring results was *r*_S_ = − 0.319 (*n* = 57; *p* = 0.016). The correlation with the naphthalene concentration in the air recorded on Thursday was *r*_S_ = − 0.280 (*n* = 57; *p* = 0.035), and with the exposure index *r*_S_ = − 0.256 (*n* = 57; *p* = 0.054).

**Table 5:** Cross-week changes of CC16 in blood serum and cellular and humoral parameters of nasal lavage fluid, comparison of study groups (median; min–max)

	Time	Reference (*n* = 12/22^a^)	Moderately exposed (*n* = 8/17^a^)	Highly exposed (*n* = 14/22)
CC16 (ng/mL)	Monday	8.6 (4.3–25.0)	8.0 (4.1–12.4)	7.8 (3.8–13.0)
	Thursday	7.8 (4.7–18.2)	6.7 (3.6–12.3)	5.8 (4.0–11.0)
Total cell count (× 10^4^)	Monday	4.6; 4.4–23.2	4.6; 4.4–67.2	6.7; 4.4–32.5
	Thursday	4.6; 4.4–22.5	8.9; 4.4–27.0	8.9; 4.4–45.6
Neutrophils (× 10^3^)	Monday	25.3; 1–86	30.0; 1–93	23.3; 0.5–96
	Thursday	31.0; 3.5–90	36.0; 1–77	21.3; 1.5–77.5
Epithelial cells (× 10^3^)	Monday	80.3; 14–100	70.5; 6.5–100	83.5; 4–100
	Thursday	78.3; 10–100	64.5; 23.0–100	83; 22.5–100
Total protein (µg/mL)	Monday	53; 10–124	34; 10–152	26; 10–338
	Thursday	38.5; 10–76	43; 10–154	32.5; 10–184
IL-8 (pg/mL)	Monday	352; 3–3931	217; 3–1227	168; 17–2034
	Thursday	209; 3–1601	188; 3–1329	235; 16–2451
MMP-9 (ng/mL)	Monday	38; 0.8–151	19; 0.03–165	22; 0.03–603
	Thursday	23; 0.5–197	43; 0.03–452	55; 0.03–613
TIMP-1 (ng/mL)	Monday	11.0; 0.3–38.6	6.0; 0.03–33.7	5.5; 0.03–29.7
	Thursday	9.7; 0.6–27.3	7.2; 0.03–36.8	6.5; 0.03–43.8
LTB_4_ (pg/mL)	Monday	32.9; 18–407	27.8; 16–205	33.0; 14–689
	Thursday	39.0; 15–200	28.3; 12–237	40.9; 12–306
8-Iso-prostane (pg/mL)	Monday	140.8; 96.7–447.7	135.0; 44.5–290.1	143.9; 63.4–363.9
	Thursday	125.9; 71.7–290.4	142.3; 59.8–317.4	153.0; 79.3–415.5
Substance P (pg/mL)	Monday	30.0; 9.8–86.2	28.1; 12.0–42.6	20.2; 13.3–40.0
	Thursday	30.5; 14.3–69.1	23.5; 14.4–50.2	24.0; 11.4–56.7

^a^Missing values were below limit of quantification (LOQ)

Cellular and humoral parameters in NALF and IS are summarized in Tables 5, 6 with the exception of IL-6 which was below the limit of detection in more than 50% of the collected serum, NALF and IS samples (data not presented). In NALF, the total cell count, the epithelial cell number, and the level of neutrophils did not differ by exposure and showed no changes during the study week. Concentrations of total protein, 8-Iso-prostane, LTB_4_, Substance P, IL-8, MMP-9 or TIMP-1 were unaffected and no statistically significant exposure and cross-week effects were observed. Similar to NALF, no differences could be observed for the measured cellular and humoral parameters in IS, neither between the three groups nor between Monday pre-shift and Thursday post-shift. The data also did not indicate an exposure-dependent increase of CRP.

**Table 6:** Cross-week changes of cellular and humoral parameters of sputum samples, comparison of study groups (median; min–max)

	Time	Reference (*n* = 12/22^a^)	Moderately exposed (*n* = 8/17^a^)	Highly exposed (*n* = 14/22^a^)
Total cell count (× 10^4^)	Monday	14.9; 5.3–43.5	13.7; 4.3–73.0	7.9; 2.3–61.1
	Thursday	25.3; 3.1–51.4	8.4; 2.1–19.4	7.3; 2.1–40.4
Neutrophils (× 10^3^)	Monday	13.5; 0.5–35.5	26.5; 3.5–52.5	4.8; 0.5–71.5
	Thursday	3.5; 0.5–35	6.5; 0.5–41.5	5.3; 1.5–12
Epithelial cells (× 10^3^)	Monday	93.8; 55.5–100	94; 43.5–100	97.5; 22.5–100
	Thursday	94.5; 33.5–100	95.8; 53.5–10	99.3; 76–1006
Total protein (µg/mL)	Monday	201.5; 69–465	144; 52–1035	136; 13–1533
	Thursday	262.5; 121–477	128.5; 35–354	140.5; 15–701
IL-8 (pg/mL)	Monday	671; 16–9379	567; 26–35,270	518; 11–49,795
	Thursday	1083; 276–8414	576; 22–2.856	355; 16–16,847
MMP-9 (ng/mL)	Monday	184; 0.2–796	42; 0.9–2.336	104; 0.4–2.296
	Thursday	237; 0.2–1193	106; 9.0–285	26; 0.7–1.695
TIMP-1 (ng/mL)	Monday	18.8; 1.5–38.9	11.4; 0.4–169	15.1; 0.03–184
	Thursday	20.1; 6.4–87.4	9.1; 0.03–27.5	7.9; 0.03–102
LTB_4_ (pg/mL)	Monday	465; 52–2698	292; 42–2790	528; 54–5063
	Thursday	766; 91–1856	189; 42–1295	423; 35–3850
8-Iso-prostane (pg/mL)	Monday	318; 6–5897	166; 19–1456	452; 19–3138
	Thursday	221; 6–3411	128; 101–1862	308; 16–3385
Substance P (pg/mL)	Monday	45.6; 9.8–169.1	34.6; 9.8–88.6	45.3; 9.8–163.0
	Thursday	28.6; 9.8–234	46.4; 33.9–145	52.1; 9.8–228

^a^Missing values were below limit of quantification (LOQ)

## Discussion

This cross-sectional study was conducted at workplaces in the manufacture of abrasive materials where naphthalene is used in open processes as a pore-forming compound and is the most important hazardaous substance. The goal was to evaluate the irritative and inflammatory effects of naphthalene on the eyes and the airways in exposed workers by using a series of various enpoint measures including self-reported work-related complaints and intensities of odor and irritation perception, nasal endoscopy, smell sensitivity, and inflammatory markers in serum, NALF and IS. A previous report on this cohort revealed that naphthalene in air was high and, specifically in working areas with direct handling of naphthalene, short-term peak concentrations up to 145.8 mg/m^3^ could be observed (Weiss et al. 2020).

The results were compared to controls who were working in spatially separated parts of the manufacturing areas or in separate office buildings. It was not possible to find controls without any naphthalene exposure at all due to the wide transmission of naphthalene in these plants. Nevertheless, as the naphthalene concentrations in the three exposure groups differed by at least two orders of magnitudes, the assessment of exposure-dependent effects was possible.

The odor of naphthalene was assessed as intense and unpleasant by the workers. There were no obvious habituation effects. While handling naphthalene directly (e.g., sieving pure naphthalene), the exposed subjects described acute sensory irritating effects at the nose and the eyes during work. Once exposure stopped and workers left their workplace they did not report any acute sensory irritating effects anymore.

In the general population, there is a percentage of approximately 20% hyposmia/anosmia (Desiato et al. 2020) which is largely due to an increased prevalencce of olfactory loss with aging (Oleszkiewicz et al. 2019). In our study the number of employees with a decreased olfactory function (21%) has therefore not exceeded the expected numbers.

With regard to nasal inflammation, the degree of reddening and swelling of the nasal mucosa and abnormal mucus production (serous or purulent secretion), represented by the total endoscopic score, have been rated as only slight to moderate by ENT specialists and compared to the controls.

Our results showed that the serum concentration of CC16, a sensitive biomarker of lung injury (Heldal et al. 2013), was lower on Thursday post-shift compared to Monday pre-shift. This was seen in all three exposure groups, but with a more pronounced effect in the highly exposed group. Although the CC16 levels did not differ significantly between the three groups, neither on Monday pre-shift nor on Thursday post-shift, the values tend to be lower in the highly exposed group. CC16 is released by epithelial cells into the serum and is a result of acute exposures to chemicals. In contrast, at chronic exposures with subsequent tissue damage, the concentrations tend to be low. In example, decreased CC16 in serum have been reported in chronic exposure to cigarette smoke after smoke-induced Club cell toxicity (Hermans and Bernard 1999; Raulf et al. 2017). However, the interpretation of slightly lower CC16 levels in workers highly exposed to naphthalene should be cautioned because CC16 levels are controlled by various factors and conditions rather than exposure alone (LaKind et al. 2007). In example, CC16 in serum is influenced by, among others, its production rate by Club cells, the permeability of the pulmonary epithelial barrier (diffusion rate from NALF into serum) and its renal clearance by glomerular filtration (LaKind et al. 2007).

Overall, although there was an indication of mild inflammatory changes, no consistent pattern of (inflammatory) effects was seen, neither in the moderately nor in the highly exposed group. Particularly with regard to various biomarkers, there was no difference in the cellular and mediator composition between the moderately and highly exposed groups. Compared to our results, changes of concentrations of biological markers signs of inflammation) found in collectives with a known exposure to a strong irritant or toxic pollutant such as tobacco smoke, and also in collectives with known lung and/or airways’ diseases (e.g., chronic obstructive lung disease (COPD), asthma, pneumonitis, lung fibroses) are by far more striking.

One of the strengths of our study is the cross-week design, including single-shift exposure assessments, which allowed the evaluation of both (sub)chronic and acute health effects due to exposure to naphthalene. The exposed employees, with only few excpetions, worked for many years at the companies, so that any long-term effects of naphthalene could be clarified. Furthermore, all study subjects also served as their own controls as they were also examined pre-shift on Monday rather than Thursday post-shift only. Therefore, it was also possible to analyze any short-term effects intra-individually.

Annother strength of our study is the limitation of potentially confounding factors such as exposure to other chemical irritants. Naphthalene is considered the only relevant exposure in the abrasives industry. Nevertheless, we carefully evaluated the overall exposure situation at these workplaces with a particular preference on dust and on organic substances (such as phenol and aldehydes) that may also cause sensory irritation. Exposure to inhalable and respirable dust, especially from ceramic grain or silica, was possible in the mixing and sieving areas rather than naphthalene alone thus specifically in the group with high exposure to naphthalene. This grain is essential in the production of ceramic grinding wheels and therefore inevitable at these workplaces. In addition, specifically in the group with moderate exposure to naphthalene, exposure to dusts from hardened grinding wheels was possible. However, these particles were neither water-soluble nor chemically reactive. It is noteworthy that the majority of particles according to their mass concentrations measured was inhalable but not respirable. Past measurements by air sampling in three companies revealed low exposures to respirable dust (≤ 1.0 mg/m^3^). In addition, crystalline silica at low concentrations (≤ 100 µg/m^3^) was only detected in two companies. We also examind the results of the clinical examinations and of the biological markers specifically for one plant where no polymer grinding wheels were produced. The results did not differ from those of the total cohort. Finally, former company measurements of phenol and formaldehyde, substances that may cause sensory irritantion, showed concentrations below current occupational exposure limits in Germany (formaldehyde: 0.3 ppm; phenol: 2 ppm). All in all, the influence of dust and other well-known sensory irritants is considered of minor relevance for the outcome of our study thus confirming naphthalene of being the primary exposure source in our collective.

Finally, we carefully checked additional confounding factors which might have influenced the outcome of our study. This included, among others, work activities at home (e.g., painting or welding), the presence of acute colds or other airways diseases, and other critical medical conditions which may influence sensory irritation and nasal inflammation. In addition, outliers (in terms of extreme values) were checked for whether the respective subject showed any distinctive feature or for methodological errors. None could be found.

The major limitation of our study is the small number of participants. This limited number was a direct result of our primary effort to aim for study participants who are almost exclusively exposed to naphthalene. Such ‘mono-exposures’ are rare to find even within the industrial sector of abrasive materials. Naphthalene is only used in the production of high-quality abrasives which, in turn, is only carried out by few companies in Europe with a total workforce of about 200 employees. In addition, it was necessary to carry out our studies in non-smokers only.

Another shortcoming which might influence the results is a ‘healthy-worker effect’, i.e., individuals who are susceptible to acute and (sub)chronic effects of sensory irritants such as naphthalene have left the workplace prematurely, whereas those who are less sensitive (or insensitive) remain. Indeed, our results show that employees in the highly exposed group reported themselves as slightly less sensitive to odors and chemicals compared to those in the other two groups. However, according to the information provided by the company doctors, the overall fluctuation of the workforce was extremely low. In addition, olfactory fatigue could not be observed in those participants regularly exposed to naphthalene suggesting that, in contrast to rodents (West et al. 2003), tolerance does not mask relevant effects in our study group. Consequently, no significant ‘healthy-worker effect’ in our study could be verified.

In summary, because reliable results from epidemiologic studies were lacking, a cross-sectional study at workplaces in the abrasives production where naphthalene is the only relevant chemical exposure was conducted. Our results in humans suggest exposure-related eye and nasal complaints due to naphthalene in the highly and moderately exposed group. Sensory irritation and odor perception (“stench”) was almost exclusively limited to the highly exposed group and associated with direct exposures to naphthalene while mixing and sieving. In contrast, no consistent pattern of nasal or pharyngeal irritant and inflammatory effects could be observed, neither with nasal endoscopy nor with inflammatory markers in serum, NALF and sputum.
